# Biocontrol potential of endophytic *Trichoderma harzianum* AUMC 14897 against *Fusarium* seedling blight disease in oat

**DOI:** 10.1186/s12870-025-06517-7

**Published:** 2025-05-05

**Authors:** Nessma A. El Zawawy, Mohamed A. El-Esawi, Nadia Attia, Yehia A.- G. Mahmoud

**Affiliations:** Botany Department, Faculty of Science, Tanta University, Tanta, 31527 Egypt

**Keywords:** Oats, *Fusarium oxysporum*, Seedling blight, Mycoparasitism, *Trichoderma* biocontrol agent

## Abstract

**Background:**

Oat (*Avena sativa* L.) represents one of the important cereal crops grown in different areas around the world due to its use in human nutrition, food industry, biomaterials, and pharmaceutical industries. *Fusarium* seedling blight disease (FSBD) represents one of the most dangerous diseases affecting oat cultivation. Endophytic fungi proved to be useful in plant disease management. Therefore, the present study investigated the impact of applied endophytic *Trichoderma harzianum* AUMC 14897 culture filtrate (CF) on disease severity, plant performance, defense systems, antioxidant activity, and stress-related genes expression in oat plants infected with *Fusarium oxysporum*.

**Results:**

The dual culture assay results revealed that *T. harzianum* is antagonistic against *F. oxysporum* and could inhibit the growth by 86.6% seven days post inoculation. Scanning electron microscope results showed that the antagonism mechanisms include nutrition, space competition, and mycoparasitism. GC–MS analysis demonstrated the presence of several volatile organic compounds in *T. harzianum* CF and each component might contribute to its biological activity. In a greenhouse experiment, spraying and irrigation with *T. harzianum* CF revealed less severe symptoms and slower disease development in the infected oat plants compared to untreated plants. Moreover, *T. harzianum* CF treatment significantly enhanced the levels of total reducing power, phenolics, flavonoids, chlorophyll, carotenoids, antioxidant enzymes, and stress-related genes expression in *F. oxysporum*-infected oat plants.

**Conclusions:**

Our results demonstrated *T. harzianum* CF has an effective role in controlling FSBD in oat plants as a novel biocontrol agent.

**Supplementary Information:**

The online version contains supplementary material available at 10.1186/s12870-025-06517-7.

## Background

Oats, known as *Avena sativa* L., represent one of the most widely grown cereal crops in different regions [1]. Oats are most commonly used as animal feed, but they also have significant roles as a raw material in the food industry as well as in the pharmaceutical and biomaterials industries [2]. Oats have been cultivated for a long time and play an important role in human nutrition [3, 4]. Oat is a healthy choice due to its balanced nutritional profile, dietary fiber and D-glucans, which can be found in high concentrations [5].

Fungal diseases are a major concern for cereal cultivation [6]. *Alternaria* species are pathogens which infect cereal plants worldwide [7, 8]. Moreover, the most common causes of *Fusarium* head blight, seedling blight, and rot in small grain cereals are *F. graminearum*, *F. culmorum*, and *F. oxysporum* [9, 10]. The extensive dissemination of these pathogens restricts the presence of host resistance sources. The potential for food and feed contamination by mycotoxins contributes to global crop losses with significant economic implications [11–13]. *Fusarium* blight disease causes destructive impacts on oats worldwide [14, 15]. This pathogen has received more attention over the years due to its severe damage to the host [16].

Chemical fungicides have been previously applied to control fungal plant diseases [17]. However, these chemical fungicides have negative impacts on the environment and public health. Therefore, searching for eco-friendly approaches to control seedling blight in oats is of utmost importance. There is strong evidence suggesting that endophytes have a significant effect on the productivity of plants in both natural and agro-environmental conditions [18]. Endophytic fungi have been shown to be useful for disease management in many plants through multiple potential mechanisms where they can enhance plant growth and development, including interference with seed germination [19]. Endophytes have numerous benefits for plants, including their role in nitrogen fixation, thermal protection, and resistance to environmental stress [20, 21]. Endophytic fungi have been extensively used as biofertilizers [21]. To the best of our knowledge, little is known about using the culture filtrate (CF) of endophytic *T. harzianum* AUMC 14897 for the control of *Fusarium* seedling blight disease (FSBD) in oats. Therefore, the main objective of this research was to examine the impact of endophytic *T.* *harzianum* CF on the severity of FSBD in oats. The current study also assessed the impact of *T.* *harzianum* CF on the infected oat plant performance, defense systems, and stress-related genes expression.

## Materials and methods

### Causal pathogen and antagonist

*Trichoderma harizanum* AUMC 14897 strain was investigated as a fungal antagonist and was deposited in NCBI GeneBank with accession No. MZ025966. This isolate was previously isolated and identified as an endophytic fungus from cladodes of *Opuntia ficus-indica* (L.) Mill. (Cactaceae) [22]. *Fusarium oxysporum* AUMC 2403 strain isolated from the soil was obtained as a pathogen strain from Assiut University Mycological Centre (AUMC).

Strains were cultured on petri-plates containing potato dextrose agar amended with chloramphenicol (300 µg/mL) to avoid the bacterial growth for 7 days at 30 °C. After incubation, culture purity of both strains was assessed and micro-morphological characteristics were tested using a light microscope (Olympus cx51, Japan), as previously described [23].

### Antagonistic potential of *T. harzianum *against* F. oxysporum *in vitro

#### Dual culture assay

The dual culture test [24] was used to examine the antagonistic potential of *T. harzianum* against *F. oxysporum *in vitro. Mycelial discs (0.5 cm in diameter) of *F. oxysporum* and *T. harzianum* were inoculated on opposite sides of a PDA plate, while *F. oxysporum* and *T. harzianum* were inoculated alone as controls. Three replicates of each treatment were maintained and incubated at 30 °C for 7 days before their antagonistic potential was evaluated. Mycelial growth of pathogen was documented, and the percentage of inhibition compared to the control was determined. The formula for determining the percentage of radial growth inhibition (RGI) is as follows:

$\%\;RGI\;=\;(C\;-\;T)\;/C\times100$, where C is the examined pathogen radial growth without the antagonist, and T is the examined pathogen radial growth in dual culture with the antagonist [25].

#### Dual-cultural interaction zones depicted by a scanning electron microscope

According to Nofal et al. [26], the interaction zone between *F. oxysporum* hyphae and *T. harzianum* was studied using a scanning electron microscope (SEM). Samples were tested for mycoparasitism and photographed using the JSM-IT200 SEM series (JEOL Ltd., Tokyo, Japan).

### Preparation of pathogen inoculum and culture filtrate (CF) of antagonist

*F. oxysporum* pathogen was grown separately on PDA plates at 30 °C for 7 days. According to El-Komy et al. [27], a sterile water was added to the plates after incubation, and the conidia were then scraped. Fungal hyphae were removed from the spore suspensions, and a hemocytometer was used to measure and adjust the concentration of conidial suspension to 5 × 10^6^ conidia mL^−1^.

To prepare culture filtrate (CF) of *T. harzianum,* eight to ten mycelial discs (0.5 cm in diameter) were placed (in floating positions) in PDB-containing flasks (100 mL), and the flasks were kept in a shaker at 30 °C for 8 days. The resulting suspension was filtered and centrifuged at 10,000 rpm for 10 min at 4 °C. The supernatant served as CF and was kept at 4 °C for further use [28].

### Assessment and characterization of volatile compounds in CF of *T. harzianum* by gas chromatography–mass spectrometry analysis (GC–MS)

Metabolites were extracted from *T. harzianum* CF using the methodology indicated by Stracquadanio et al. [29]. *T. harzianum* CF was extracted thrice with ethyl acetate solvent (1:1). To dry the mixed fraction, MgSO_4_ was utilized and allowed to evaporate at 35 °C under pressure. The red-brown residues were collected and dissolved in 10% methanol (CH_3_OH). GC–MS analysis was then assayed to identify the active biomolecule constituents in the extracts. The volatile compounds in *T. harzianum* CF were chemically identified by comparing mass spectra to a library in the NIST database [30]. The temperature of the ion source was adjusted to 250 °C, and the electron impact energy was 70 eV. The mass scan (m/s) range for electron impact (EI) was 40–450 Da.

### Use of *T. harzianum* as a biocontrol agent against FSBD in oats under greenhouse conditions

#### Plant material and greenhouse experimental design

In order to examine the *T. harzianum* potentiality to suppress *F. oxysporum* growth, a controlled greenhouse experiment was conducted at the Faculty of Science, Tanta University, Egypt. Oat (*Avena sativa* L.) cv. Belinda seeds were obtained from Shandawel Research Station, Agricultural Research Center (ARC), Egypt. Seeds were sterilized with sodium hypochlorite (1%) for 30 min, washed five times in sterile water, and then planted in pots containing pasteurized sandy clay soil. Regular irrigation was provided to the seedlings throughout the experiment. The average temperature and humidity were 25 ± 2 °C and 70 ± 5%, respectively.

#### Artificial inoculation and management of FSBD by *T. harzianum*

The spraying method was used for the artificial inoculation [31]. Artificial inoculation with an inoculum suspension of *F. oxysporum* (5 × 10^6^ conidia mL^−1^) was performed by spraying each oat panicle with 1 mL of *F. oxysporum* suspension. After inoculation, polyethylene bags were placed over the panicles for 48 h to keep them moist for pathogen establishment.

The antagonistic potential of *T. harzianum* was examined in vivo by setting up a randomized complete block design for the planted pots. The pots were divided into 4 main groups: The first group represents the healthy plants with no treatment (negative control, H), and the second group was infected with a fungal suspension of *F. oxysporum* (positive control, T_0_). In the third group, *T. harzianum* spraying (T_1_) (1 mL of CF per panicle) was applied to the pots representing the positive control after two weeks of inoculation. In the fourth group, additional pots serving as a positive control were irrigated with *T. harzianum* (20 mL of CF per pot) (T_2_). With a consistent irrigation every three days for two weeks, all the plants were left to grow. The frequency of FSBD was then assessed across all pots for three consecutive weeks.

#### Disease assessment

Disease incidence (DI) was computed as indicated by Gashaw et al. [32] using the formula:

Disease incidence (%) = (a/A) × 100, where “a” is the number of diseased plants, and “A” is the total number of examined plants.

#### Physio-biochemical analyses

Total phenolic content of plant extract was determined using Folin-Ciocalteu methodology as reported by Miliauskas et al. [33] with slight modifications. Briefly, 1 mL of the extract and 5 mL of Folin-Ciocalteu solution were mixed together and kept at 25 °C for 7–10 min. Approximately, 4 mL of 7% Na_2_CO_3_ was then poured into the mixture and kept for 90–120 min at 25 °C. Absorbance was then read at 765 nm. Gallic acid equivalent (GAE) was employed to determine the total phenolic content. Total flavonoids content of the plant extracts was estimated using the methodology reported by Nongalleima et al. [34]. Quercetin was employed for determining the total flavonoid content. Moreover, the total reducing power (TRP) was estimated as previously reported [35, 36]. DPPH radical scavenging activity of the plant extracts was estimated using the methodology indicated by Thaipong et al. [37].

Chlorophyll and carotenoid contents in plant leaves were estimated as reported by Lichtenthaler and Wellburn [38]. Absorbance was measured at 663, 645, and 453 nm. Acetone (80%) was used as a blank. Superoxide dismutase (SOD) activity was quantified using nitro blue tetrazolium photoreduction, and peroxidase (POD) activity was assayed using guaiacol colorimetry. Furthermore, the method of Aebi [39] was utilized to determine catalase (CAT) activity.

#### Transcriptional analysis

Quantitative real time-PCR assay was employed to estimate the expression of stress-related genes including *ascorbate peroxidase 1* (*APX1*), catalase 1 (*CAT1*), *copper–zinc Superoxide dismutase 1* (*SOD1*), *phenylalanine ammonia lyase gene* (*PAL*), and *proline biosynthetic gene* (*P5 CS1*). Total RNA was isolated from fresh oat leaves with RNeasy Plant Mini kit (Qiagen). Contaminating DNA was removed and cDNA was then synthesized utilizing Reverse Transcription kit (Qiagen, Germany). qRT-PCR was done following the manufacturer’s protocol of QuantiTect SYBR Green PCR kit (Qiagen) and the reactions were performed as reported by Kong et al. [40]. Gene specific-primers [40, 41] were used for the genes amplification. *ACTIN1* was employed as an internal reference [40] and the genes relative expression level was estimated using 2^−ΔΔCt^ method [42].

### Statistical analysis

Data analysis was performed utilizing the analysis of variance and Duncan’s multiple range test. Values are significant at *p* ≤ 0.05. SPSS version 16 (Chicago, IL, USA) was used for analysis.

## Results

### Pathogen and antagonist isolation and identification

**Fig. 1:**
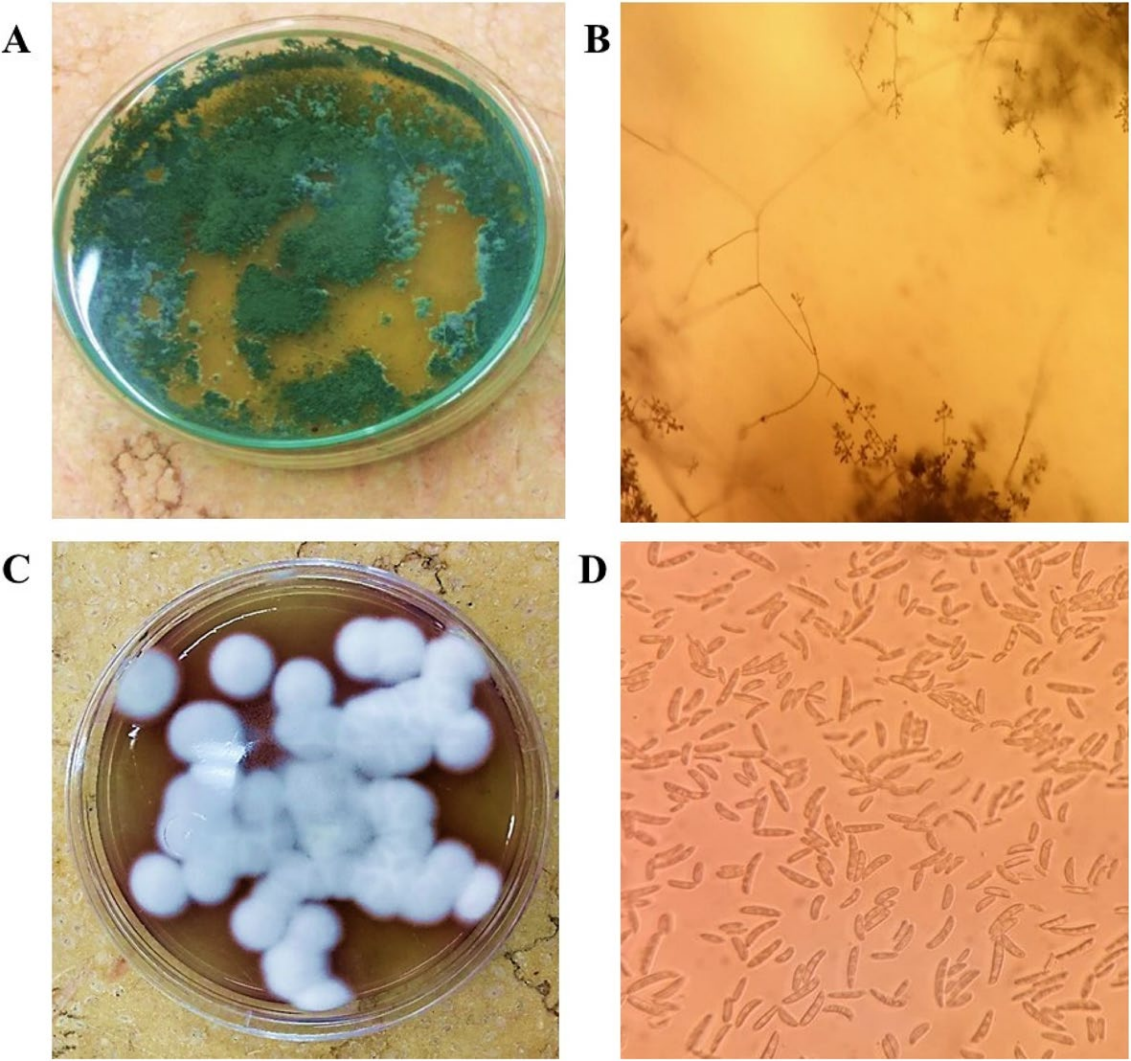
Micro-morphological examination of antagonist and pathogen. **A**, **C** Colonies of *Trichoderma harzianum* and *Fusarium oxysporum* on potato dextrose agar at 30 °C for 1 week, respectively. **B**, **D** Conidiophores and conidia at 40X magnification of *T. harzianum* and *F. oxysporum*, respectively

The antagonistic endophytic strain *T. harzianum* was assessed by characterizing its colony morphology on PDA and mycelia under a light microscope. In Fig. 1A, the colonial feature of the isolate can be seen as a single, green, and yellowish concentric ring containing a cluster of yellow conidia at the point of inoculation. In addition, the sporangia were spherical to subglobal in shape, and appeared white before turning to pale green (Fig. 1B). Meanwhile, the pathogen *F. oxysporum* appeared circular, velvety, and mottled type with grey and white colonies on PDA medium (Fig. 1C). Moreover, the macroconidia of *F. oxysporum* were numerous, long, cylindrical, slightly curved and fusiform (Fig. 1D).

### Antagonistic potential of T. harzianum against F. oxsporum by dual-culture interaction

**Fig. 2:**
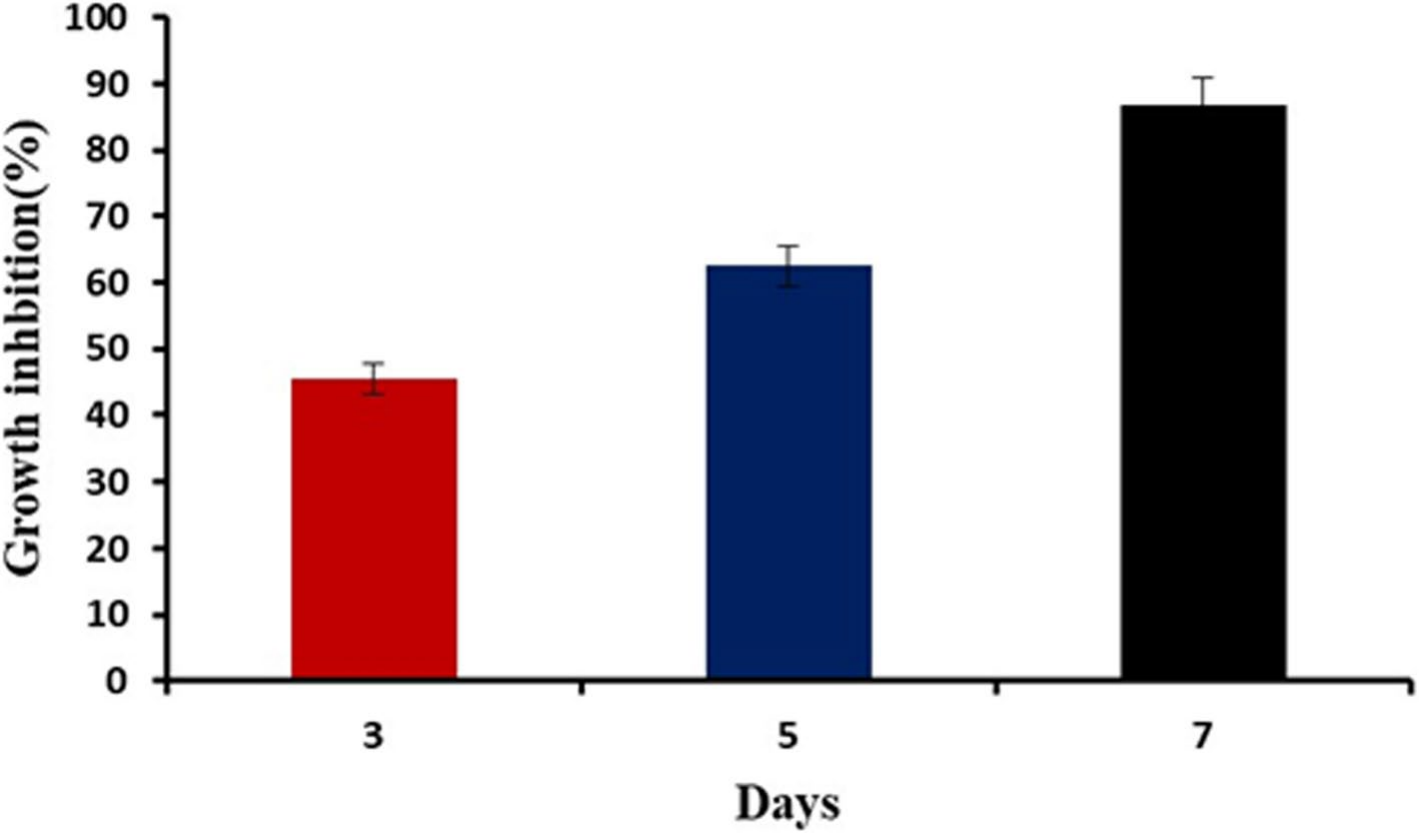
Percentage of growth inhibition of the pathogenic mycelia of *F. oxysporum* by *T. harzianum* during 7 days of inoculation

*T. harzianum* is an efficient biocontrol agent against *F. oxysporum*. *T. harzianum* inhibited *F. oxysporum* growth effectively in a dual culture assay (Supplementary Fig. 1). Five days post inoculation, *T. harzianum* inhibited *F. oxysporum* mycelial growth by 62.5%. Interestingly, *T. harzianum* was able to slow down *F. oxysporum* growth by 86.6% seven days post inoculation (Fig. 2).

### Ultra-structural changes under dual-culture interaction by SEM

**Fig. 3:**
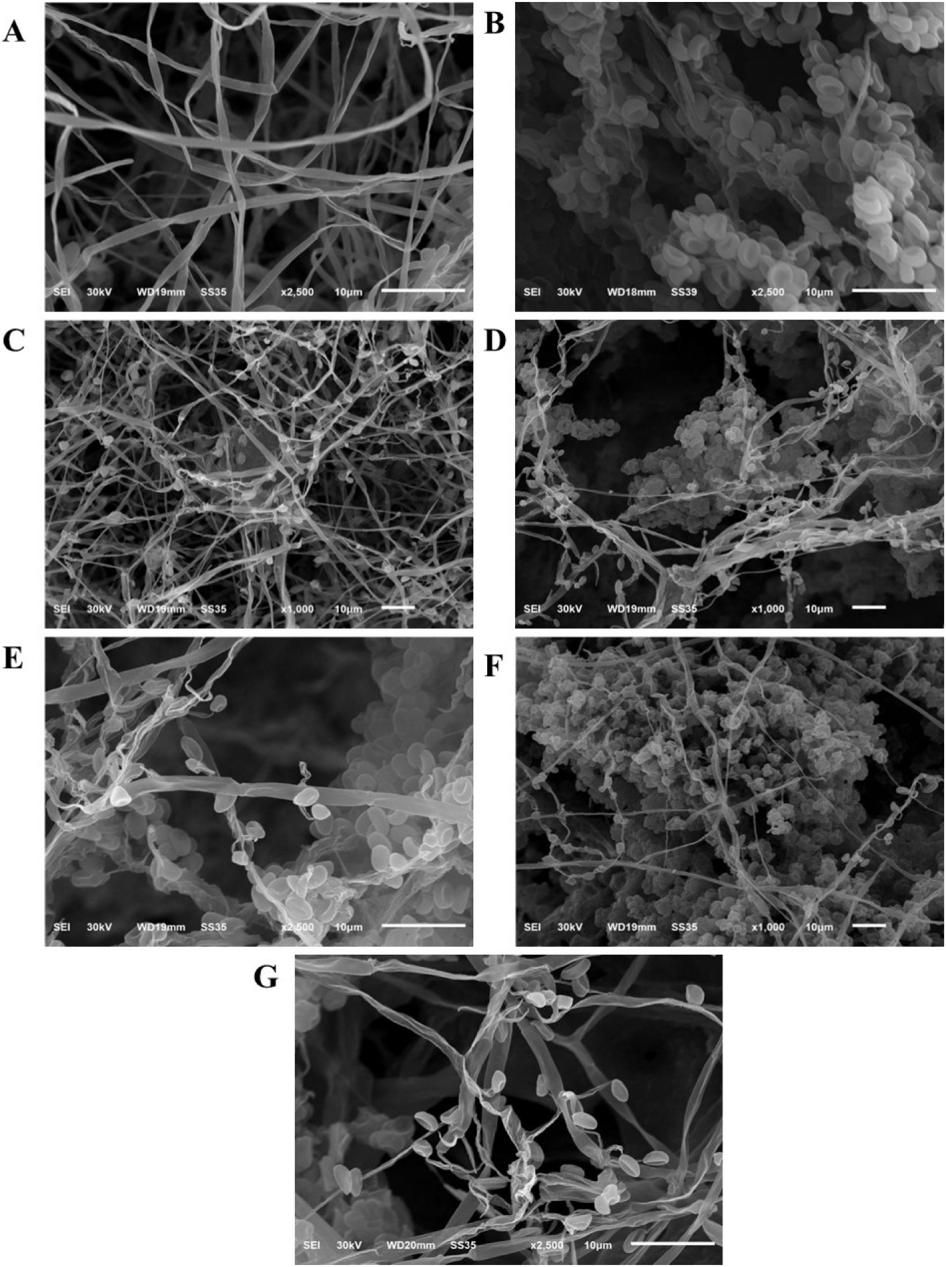
Mycoparasitic activity of *T. harzianum* against the pathogen *F*. *oxysporum*. Scanning electron microscope (SEM) images at different magnifications (1000 X and 2500 X). **A**, **B** *F. oxysporum* and *T. harzianum* inoculated alone as controls, respectively. **C** Interaction between *T. harzianum* against the pathogen *F*. *oxysporum* after 3 days. **D**, **E** Mycoparasitic activity of *T. harzianum* against *F*. *oxysporum* after 5 days showing that *T. harzianum* hyphae coiled or attached to *F. oxysporum.* **F**, **G** Mycoparasitic activity of *T. harzianum* against *F*. *oxysporum* after 7 days causing degradation and deformation of *F. oxysporum* hyphae

The mycoparasitism of *T. harzianum* against the pathogen *F. oxysporum* was evident in images taken with a scanning electron microscope at 1000 × and 2500 × magnifications. Figure 3A and B showed higher growth of both *F. oxysporum* and *T. harzianum* mycelia, which were inoculated alone as controls, respectively. After three days, *T. harzianum* and *F. oxysporum* interacted, as shown in Fig. 3C. On the other hand, after 5 days of incubation, the dual culture assay results showed that *T. harzianum* had overgrown and heavily sporulated on the colony of the pathogen *F. oxysporum* (Fig. 3D and E). After 7 days of inoculation, *T. harzianum* exhibited mycoparasitic appearance, as shown in Fig. 3F and G, in the forms of coiling adhesion, penetration, and deformation. Based on scanning electron microscopy results, the antagonist mechanisms of *T. harzianum* towards the pathogen *F. oxysporum* are nutrient and space competition, mycoparasitism, and antibiosis.

### GC–MS analysis of *T. harzianum* culture filtrates (CF)

The GC–MS chromatogram demonstrated the presence of several volatile compounds in *T. harzianum* CF, as shown in Table 1 and Supplementary Fig. 2. Numerous compounds containing hydroxyl groups, aldehydes, hydroxyls, various acids, and esters were identified in *T. harzianum* CF. At a retention time (RT) of 26.66, n-Hexadecanoic acid was found to be the most abundant compound in *T. harzianum* CF (41.77%) followed by heptadecane (20.29%) and other compounds at lower concentrations.

**Table 1:** GC-MS analysis of the volatile compounds from *T. harzianum* CF

**Peak no.**	**RT**	**Molecular formula**	**Molecular weight**	**Compound name**	**Area%**	**Structure**
1	6.238	C_9_H_18_O	158.238	2-Heptanol, acetate	0.466	
2	6.984	C_14_H_24_O_2_	224.3392	1,5-Dimethyl- 1-vinyl- 4-hexenyl butyrate	0.421	
3	8.344	C_9_H_12_	120.1916	Benzene, 1-ethyl- 2-methyl-	1.001	
4	8.514	C_9_H_12_	120.1916	Benzene, (1-methylethyl)-	1.081	
5	9.145	CH_3_NO_2_	61.0400	Methane, nitro-	0.442	
6	9.625	C_10_H_20_O_2_	172.26	2-Propenoic acid, 2-methyl-, hexyl ester	0.420	
7	10.27	C_12_H_26_	170.33	Heptane, 2,2,4,6,6-pentamethyl-	0.800	
8	11.836	C_12_H_12_O_06_	252.22	Benzene, 1,3,5-trimethyl	2.665	
9	12.986	C_13_H_28_	184.37	Tridecane	0.576	
10	13.136	C_12_H_26_	170.33	Heptane, 5-ethyl- 2,2,3-trimethyl-	0.374	
11	14.107	C_10_H_14_	134.22	1,3,8-p-Menthatriene	0.387	
12	14.637	C_11_H_24_	156.31	Undecane	1.611	
13	15.057	C_10_H_14_	134.22	Benzene, 1,2,4,5-tetramethy	0.508	
14	16.688	C_8_H_18_O	130.23	3-Octanol	0.632	
15	17.183	C_4_H_6_S	86.16	Thiophene, 2,3-dihydro-	0.770	
16	18.208	C_15_H_32_	212.41	Dodecane, 2,6,11-trimethyl-	2.679	
17	20.969	C_26_H_54_	366.7	Octadecane, 3-ethyl- 5-(2-ethylbutyl)-	0.424	
18	21.309	C_14_H_30_	198.39	Decane, 2,3,5,8-tetramethyl-	3.634	
19	23.025	C_16_H_48_O_8_Si_8_	593.2	Cyclooctasiloxane, hexadecamethyl-	1.051	
20	23.875	C_17_H_36_	240.5	Heptadecane	20.291	
21	24.371	C_21_H_44_	296.57	Heptadecane, 2,6,10,15-tetramethyl-	1.388	
22	24.526	C_14_H_28_O_2_	228.37	Myristic acid	0.840	
23	25.421	C_19_H_38_O_2_	298.5	Oxirane, [(hexadecyloxy)methyl]	0.368	
24	25.636	C_16_H_22_O_4_	278.34	1,2-Benzenedicarboxylic acid, bis(2-methylpropyl) ester	1.932	
25	26.036	C_16_H_34_	226.44	Hexadecane	0.648	
26	26.161	C_21_H_44_	296.57	Heptadecane, 2,6,10,15-tetramethyl-	1.939	
27	26.271	C_10_H_18_O_4_	202.25	Nonanedioic acid, monomethyl ester	0.824	
28	26.666	C_16_H_32_O_2_	256.4	n-Hexadecanoic acid	41.77	
29	27.052	C_19_H_40_	268.5	Nonadecane	1.752	
30	28.237	C_20_H_42_	282.5	Nonadecane, 2-methyl-	0.960	
31	28.617	C_18_H_34_O_2_	282.5	Octadecanoic acid	4.671	
32	29.863	C_17_H_32_O_2_	268.4	7-Methyl-Z-tetradecen- 1-ol acetate	0.633	
33	30.198	C_25_H_52_	352.7	Heptadecane, 9-octyl-	0.595	
34	31.963	C_18_H_34_O	266.5	4-Octadecenal	0.406	
35	32.084	C_19_H_38_O_4_	330.5	Hexadecanoic acid, 2-hydroxy- 1- (hydroxymethyl)ethyl ester	1.1	

### Effect of *T. harzianum* against FSBD under greenhouse conditions

**Fig. 4:**
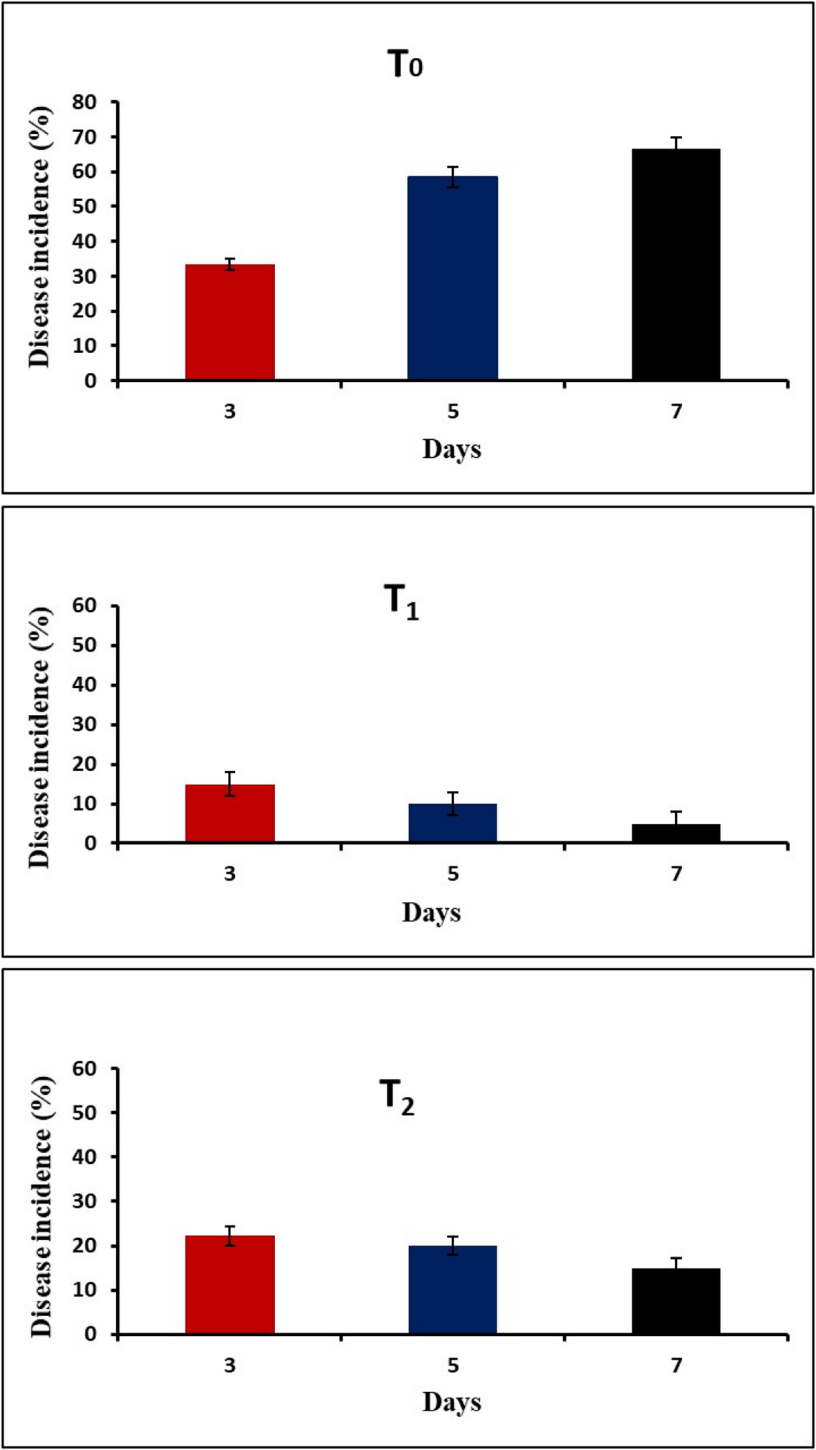
Disease incidence (%) under greenhouse condition after infection. (T_0_) Infected with *F. oxysporum*, (T_1_) Infected and treated with *T. harzianum* by spraying, (T_2_) Infected and treated with *T. harzianum* by irrigation

**Fig. 5:**
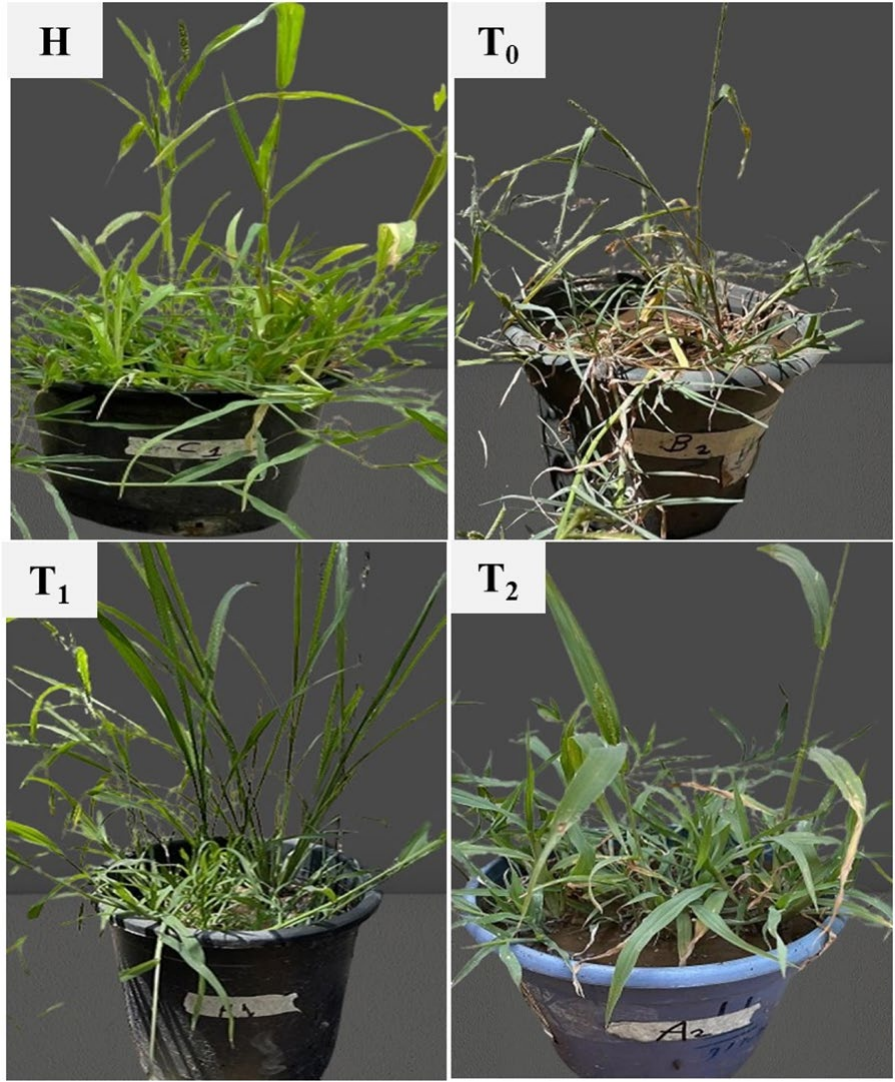
Disease incidence in oat plants, (H) Healthy plant, (T_0_) Infected plant with *F. oxysporum*, (T_1_) Infected and treated with *T. harzianum* by spraying, (T_2_) Infected and treated with *T. harzianum* by irrigation

The present study demonstrated the efficacy of *T. harzianum* as a biological control agent against FSBD incidence (%) in oat plants grown in the greenhouse. Figure 4 showed that the mean disease incidence was highest (66.6%) in *F. oxysporum*-infected oat plants (T_0_). The infected plants showed symptoms such as dwarfed seedlings, leaf yellowing, wilting, root rots, and shorter main roots. However, there were no symptoms in the healthy control plants (H) as shown in Fig. 5. Management with spraying (T_1_) and irrigation (T_2_) with *T. harzianum* showed less severe symptoms and slower disease development in infected oat plants compared to untreated plants. *T. harzianum* spraying reduced disease incidence in oat plants more effectively than irrigation (Fig. 4).

### Physio-biochemical and molecular characteristics of infected oat plants under the effect of biological control treatment

#### Total phenolics and flavonoids contents

**Fig. 6:**
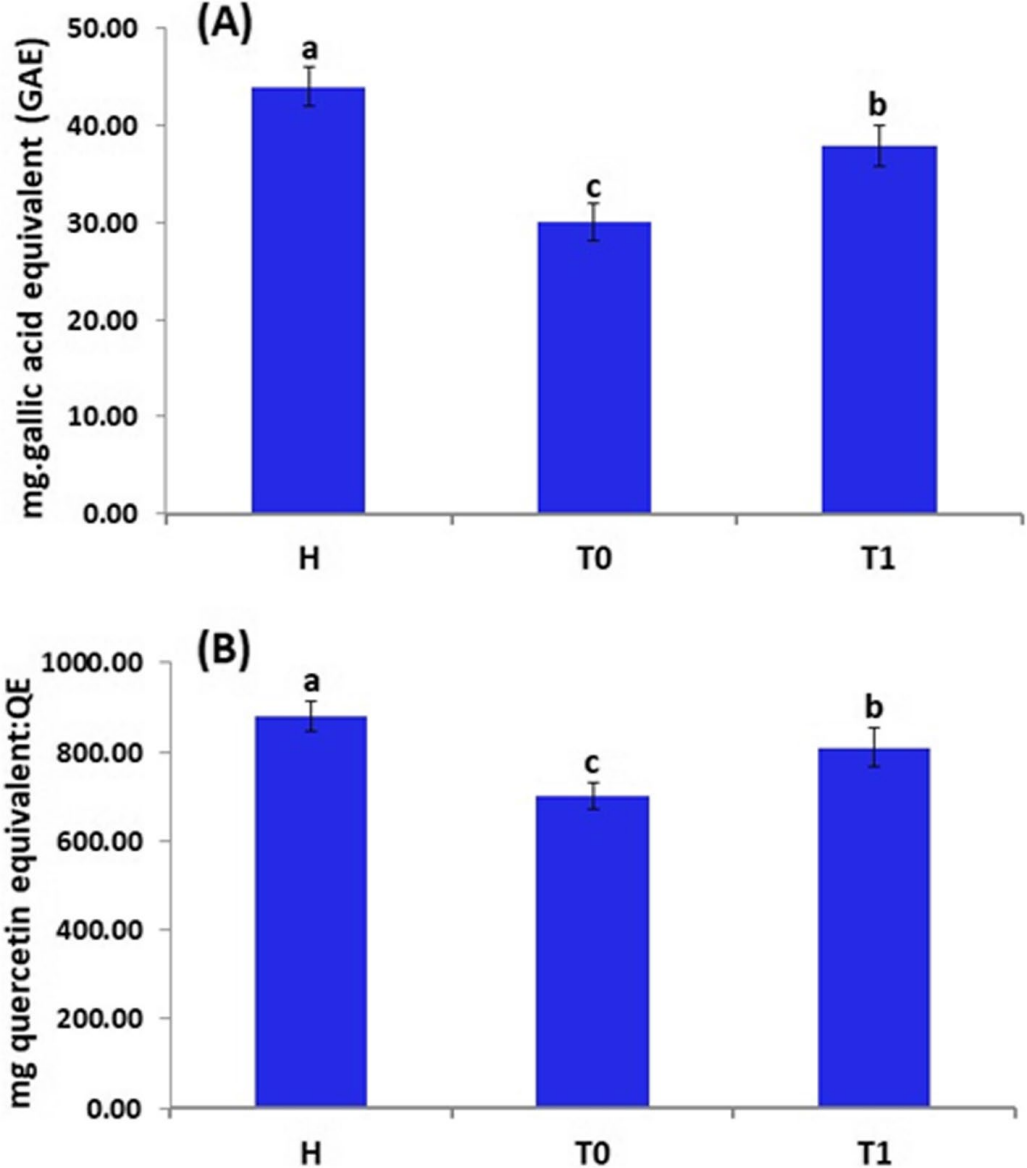
Total phenolics content (**A**) and total flavonoids content (**B**) of oat plants. (H) Healthy plant, (T_0_) Infected plant with *F. oxysporum* (control), (T_1_) Infected and treated with *T. harzianum* by spraying. Results are expressed in means ± standard error (*n* = 3). Different letters indicate significant differences at *p* ≤ 0.05

Total phenolics content in the *F. oxysporum*-infected oat plants was reduced by 32%, as compared to healthy plants. Total phenolics content in the *F. oxysporum*-infected plants treated with *T. harzianum* increased by 26.7% when compared to *F. oxysporum*-infected plants as shown in Fig. 6A.

Moreover, the total flavonoids content in the *F. oxysporum*-infected oat plants was reduced by 20.5%, as compared to healthy plants (Fig. 6B). However, the total flavonoids content in the *F. oxysporum*-infected plants treated with *T. harzianum* increased by 15.7% when compared to *F. oxysporum*-infected plants.

#### Total reducing power and radical scavenging activity

**Fig. 7:**
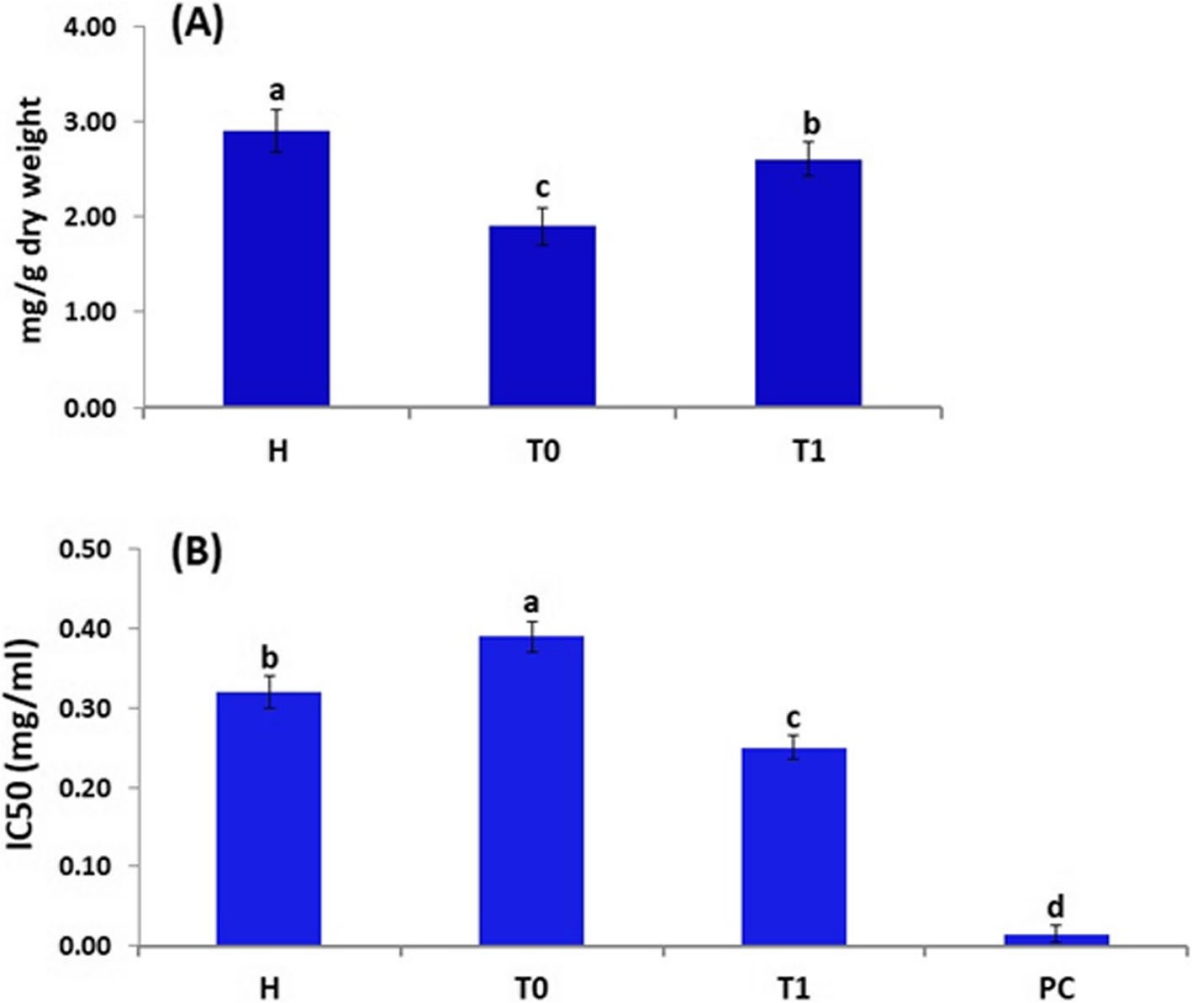
Total reducing power (**A**) and DPPH (**B**) of oat plants (H) Healthy plant, (T_0_) Infected plant with *F. oxysporum* (control), (T_1_) Infected and treated with *T. harzianum* by spraying. PC is a positive control (ascorbic acid). Results are expressed in means ± standard error (*n* = 3). Different letters indicate significant differences at *p* ≤ 0.05

Data presented in Fig. 7A showed that the total reducing power (TRP) in the *F. oxysporum*-infected oat plants was reduced by 34.5%, as compared to healthy plants. However, the total reducing power in the *F. oxysporum*-infected plants treated with *T. harzianum* increased by 36.8% when compared to *F. oxysporum*-infected plants.

Furthermore, the radical scavenging activity (DPPH) in the *F. oxysporum*-infected oat plants increased by 21.9%, as compared to healthy plants (Fig. 7B). However, the radical scavenging activity in the *F. oxysporum*-infected plants treated with *T. harzianum* was reduced by 35.9% when compared to *F. oxysporum*-infected plants.

#### Chlorophyll and carotenoids contents

**Fig. 8:**
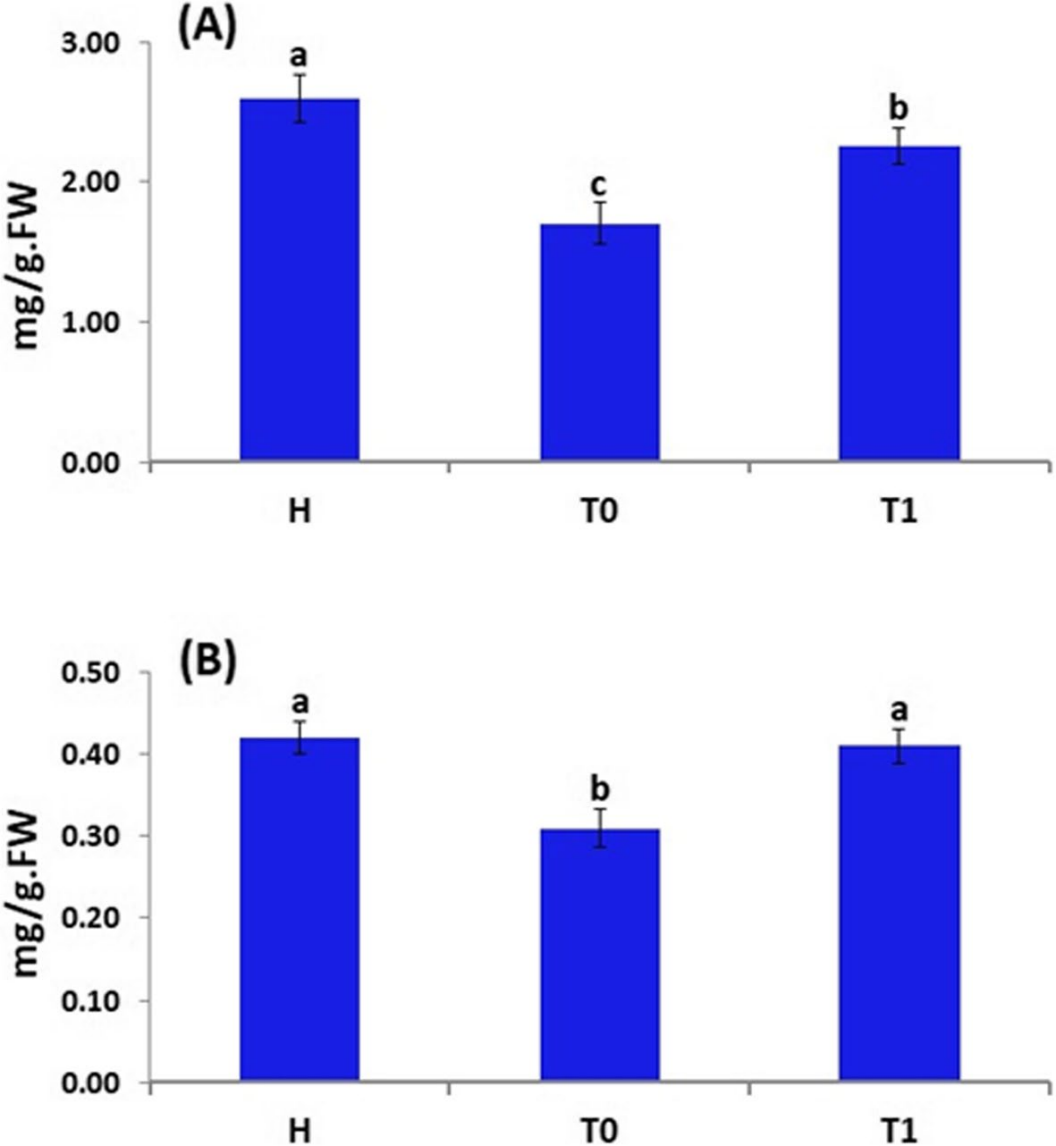
Chlorophyll content (**A**) and carotenoids content (**B**) of oat plants (H) Healthy plant, (T_0_) Infected plant with *F. oxysporum* (control), (T_1_) Infected and treated with *T. harzianum* by spraying. Results are expressed in means ± standard error (*n* = 3). Different letters indicate significant differences at *p* ≤ 0.05

The chlorophyll content in the *F. oxysporum*-infected oat plants was reduced by 34.6%, as compared to healthy plants. However, the chlorophyll content in the *F. oxysporum*-infected plants treated with *T. harzianum* increased by 32.4% when compared to *F. oxysporum*-infected plants (Fig. 8A).

Moreover, the results revealed that the carotenoids content in the *F. oxysporum*-infected oat plants was reduced by 26.2%, as compared to healthy plants (Fig. 8B). However, the carotenoids content in the *F. oxysporum*-infected plants treated with *T. harzianum* increased by 32.3% when compared to *F. oxysporum*-infected plants.

#### Antioxidant enzymes activity

The activities of SOD, CAT and POD in the *F. oxysporum*-infected oat plants increased by 31.7, 8.6 and 4.6%, as compared to healthy plants. Furthermore, *T. harzianum* treatment significantly enhanced the activities of these antioxidant enzymes in infected oat plants, as compared to the other treatments. The highest levels of antioxidant enzymes were recorded in the *F. oxysporum*-infected plants treated with *T. harzianum* (Table 2).

**Table 2:** Antioxidant enzymes activity of oat plants

Treatments	Superoxide dismutase activity (µg.g^−1^.min^−1^ FW)	Catalase activity (µg.g^−1^.min^−1^ FW)	Peroxidase activity (µg.g^−1^.min^−1^ FW)
H	30.32 ± 0.28^d^	550.31 ± 51.2^d^	611.41 ± 58.7^d^
T_0_	39.92 ± 0.37^b^	597.41 ± 58.5^b^	639.34 ± 54.7^b^
T_1_	42.65 ± 0.33^a^	611.46 ± 60.1^a^	655.39 ± 62.1^a^

(H) Healthy plant, (T_0_) Infected plant with *F. oxysporum* (control), (T_1_) Infected and treated with *T. harzianum* by spraying. Results are means ± standard error (*n* = 3). Means followed by different letters within the same column are significantly different at *P* ≤ 0.05

#### Expression of stress-related genes

Data presented in Table 3 revealed that the expression level of *SOD1*, *CAT1*, *APX1*, *PAL*, and *P5 CS1* were significantly induced in *F. oxysporum*-infected oat plants, as compared to healthy plants. Furthermore, *T. harzianum* treatment significantly enhanced the expression level of these stress-related genes in infected oat plants, as compared to the other treatments. The highest expression levels of such stress-related genes were recorded in the *F. oxysporum*-infected plants treated with *T. harzianum.*

**Table 3:** Expression levels of stress related genes in leaves of oat plants

Treatments	*Copper–Zinc Superoxide dismutase 1* (*SOD1*) gene	*Catalase 1* (*CAT1*) gene	*Ascorbate peroxidase 1 (APX1)* gene	*PAL* gene	*P5 CS1* gene
H	1.00 ± 0.12^d^	1.00 ± 0.11^d^	1.00 ± 0.13^d^	1.00 ± 0.11^d^	1.00 ± 0.10^d^
T_0_	2.21 ± 0.37^b^	1.95 ± 0.12^b^	2.41 ± 0.13^b^	2.38 ± 0.12^b^	2.54 ± 0.14^b^
T_1_	2.97 ± 0.33^a^	2.38 ± 0.13^a^	3.27 ± 0.12^a^	2.91 ± 0.14^a^	3.25 ± 0.13^a^

(H) Healthy plant, (T_0_) Infected plant with *F. oxysporum* (control), (T_1_) Infected and treated with *T. harzianum* by spraying. Results are means ± standard error (*n* = 3). Means followed by different letters within the same column are significantly different at *P* ≤ 0.05

## Discussion

Biological control approaches are considered as a promising weapon against plant pathogens. Because of its promising potential to inhibit plant pathogens, the use of endophytic fungi in agricultural systems is currently exploited as an emerging method for controlling plant diseases [43]. Endophytic *T. harzianum* CF significantly inhibited *F. oxysporum* mycelial growth when applied in vitro. Mycelial growth was reduced in the dual culture assay, demonstrating that *T. harzianum* CF could effectively inhibit *F.* *oxysporum* mycelial growth. Overgrowth and sporulation by *T. harzianum* on a colony of the *F. oxysporum* pathogen were observed 7 days after inoculation, and parallel growth by this antagonist isolate closely associated with the pathogen was also observed during this time. In addition, scanning electron microscopy revealed the mycoparastic activity of *T. harzianum* which coils around *F. oxysporum* hyphae, degrades and deforms pathogen hyphae, and grows in parallel with the *Fusarium* pathogen. These results suggest that endophytic *T. harzianum* could serve as a source of promising biological fungicides, particularly against FSBD of oat, without causing the negative effects commonly associated with chemical fungicides.

Several studies showed that *Trichoderma* spp. possess an antagonistic potential due to activating diverse biocontrol mechanisms [44, 45], which prevent several plant diseases. The current study revealed that *T. harzianum* CF has a potent inhibitory effect. Similarly, CFs of *T. simmonsii* and *T. asperellum* could inhibit the mycelial growth of *Pythium undulatum* and *Phytophthora inundata* [45]. Moreover, Alka and Prajapati [46] found that the CFs of several *Trichoderma* species, including *T. harzianum* and *T. viride*, efficiently inhibited *Rhizopus oryzae* growth. Strong inhibition of *Colletotrichum gloeosporioides* growth was also revealed by the CFs of several *Trichoderma* species when used as biofungicides [47]. Evidences from other studies demonstrated the biocontrol potential of *T.* *harzianum* by reporting a considerable mycelial growth inhibition of diverse pathogens [48]. Competition for space, food, and nutrients leads *Trichoderma* species to produce diverse metabolites that depreciate the fungal pathogens mycelia, making them effective biocontrol agents [49]. Significant inhibition of a wide variety of fungal pathogens across multiple taxonomic families has been reported by numerous *Trichoderma* species'metabolites [50, 51].

GC–MS analysis of *T. harzianum* CF showed that it produces several volatile organic compounds and each component might contribute to its biological activity. The n-Hexadecanoic acid and heptadecane were observed in relatively higher abundance percentage. Similar compounds were found in the GC–MS analysis of the CF of *Trichoderma* species previously assayed [52, 53]. Also, our results are consistent with those of Ganesan [54], Idris et al. [55] and Rahbar et al. [56] who indicated the antimicrobial activities of n-Hexadecanoic acid and heptadecane. Interestingly, the bioactive compounds may inhibit the fungus via affecting the plasma membrane, cell wall and mitochondria [57]. Additionally, our results suggest that these antifungal compounds may contribute to the biocontrol potential of *T.* *harzianum* against FSBD of oat.

The effect of the spraying application (T_1_) as a fungicide resulted in slower growth of *F. oxysporum* and better disease control than the irrigation application (T_2_) assayed in the present study. The present study also demonstrated that *T. harzianum* treatment markedly improved the levels of total reducing power, phenolics, flavonoids, chlorophyll, carotenoids, antioxidant enzymes and stress-related genes expression in the *F. oxysporum*-infected oat plants. These results indicate the effective role of *T. harzianum* in modulating the physio-biochemical and molecular processes controlling FSBD in oat plants. Our findings were in line with the results of several previous studies [58]. El-Sharkawy and Abdelrazik [58] revealed that the applications of mycorrhizal fungi and antagonistic microorganisms markedly elevated the total chlorophyll, carotenoids, total protein, free amino acids, free phenolic compounds, and antioxidant enzyme activities in *Fusarium*-infected squash plants, indicating their effective roles in controlling *Fusarium* root rot. Moreover, Bonini et al. [59] revealed that application of arbuscular mycorrhizal fungi and *Trichoderma koningii* induced secondary metabolism and accumulated phenolic compounds in pepper. Rouphael et al. [60] also demonstrated that arbuscular mycorrhizal fungi induced phenolics, photosynthetic activity, proteins, secondary metabolism and organic acids into the rhizosphere. Li et al. [61] revealed that *Trichoderma* could enhance antioxidant enzymes activity in cucumber plants.

In conclusion, the present study demonstrated the effective role of *T. harzianum* AUMC 14897 strain in controlling FSBD in oat plants through modulating the physio-biochemical and molecular processes.

## Supplementary Information

Supplementary Material 1

## Data Availability

All the data supported this study are included in this published manuscript.
