# Supplementary material for: Biocontrol potential of endophytic Trichoderma harzianum AUMC 14897 against Fusarium seedling blight disease in oat
Source: BMC Plant Biol. 2025 May 5;25:586. doi: 10.1186/s12870-025-06517-7 (PMC12051340; doi:10.1186/s12870-025-06517-7)
Supplement: Supplementary file 1 — Supplementary Material 1 [file 12870_2025_6517_MOESM1_ESM.docx]

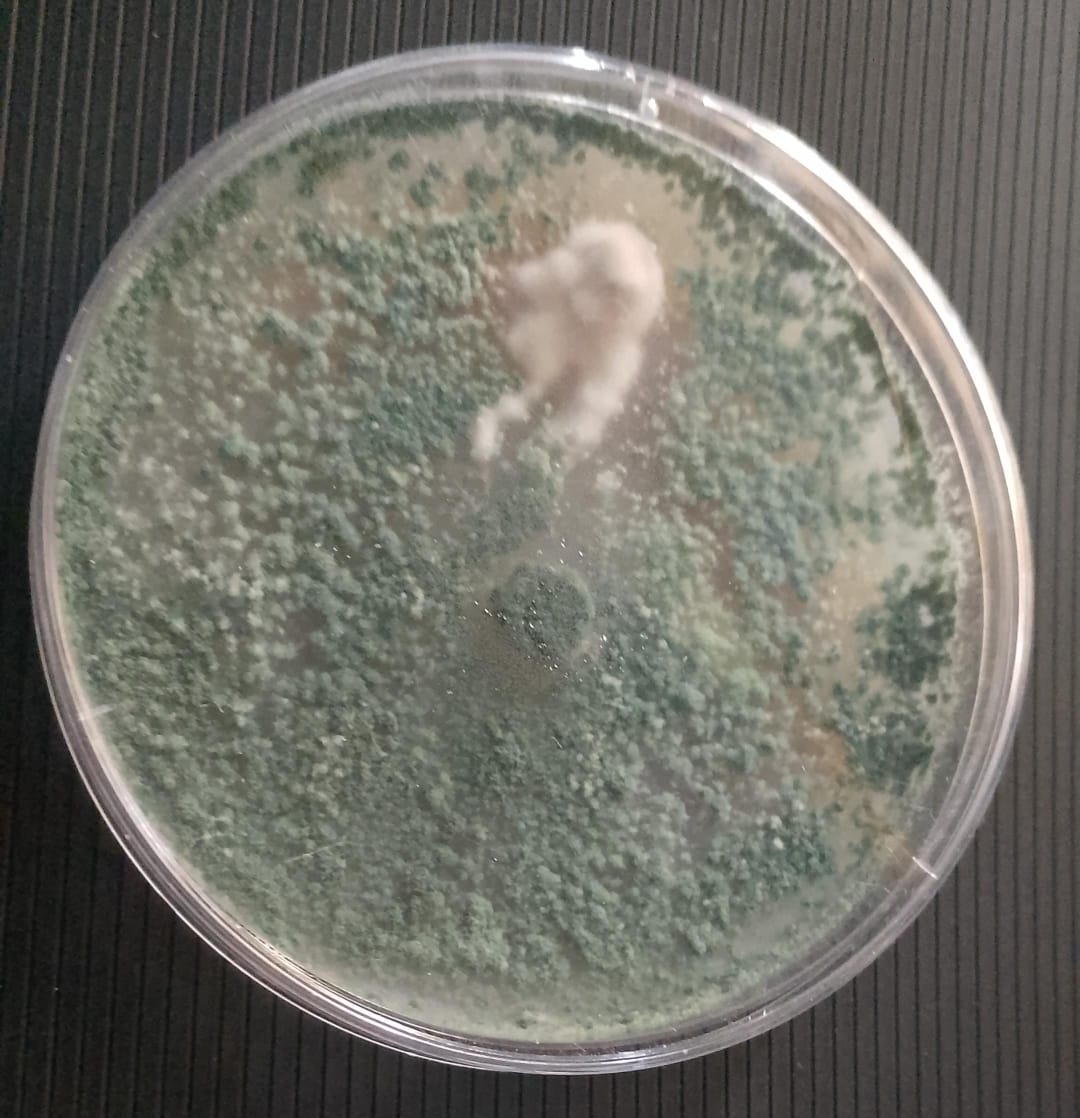


**Supplementary Figure 1.** Dual culture assay for evaluating the antagonistic activity of *T. harzianum in vitro* against *F. oxysporum*.


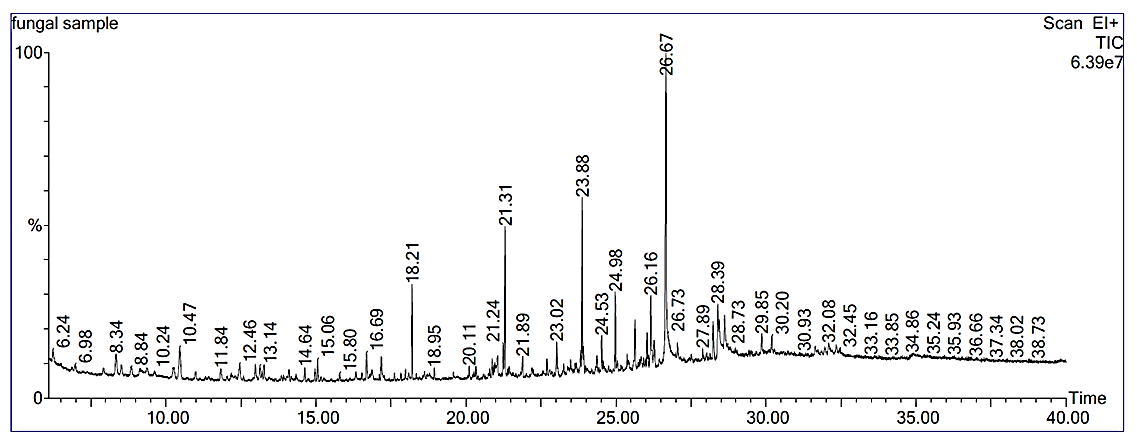


**Supplementary Figure 2.** GC-MS chromatogram of *T. harzianum* culture filtrate (CF).
